# Evaluation of the Impact of the Peregrine Falcon (*Falco peregrinus peregrinus*) Reintroduction Process on Captive-Bred Population

**DOI:** 10.3390/genes13081487

**Published:** 2022-08-20

**Authors:** Karol O. Puchała, Zuzanna Nowak-Życzyńska, Sławomir Sielicki, Wanda Olech

**Affiliations:** 1 Department of Animal Genetics and Conservation, Warsaw University of Life Sciences, 02-787 Warszawa, Poland; 2 Society for Wild Animals “Falcon”, 87-800 Włocławek, Poland

**Keywords:** microsatellites, populational genetics, *Falco peregrinus*

## Abstract

The main objective of this study was to determine the impact of increased demand for peregrine falcons via breeding (mainly Polish, Czech, German and Slovak) on the genetic structure of the birds. In the analysis, 374 specimens from six countries were sampled in 2008–2019 (omitting 2009), and all the birds analyzed were released into the wild as part of the Polish reintroduction program. The assessment of genetic variation was based on a well-known panel of 10 microsatellite markers described for the species. We calculated a fixation index for the samples from each year, and based on this, we determined the level of inbreeding. We also performed an analysis using the Bayesian cluster method, assuming that 1–19 hypothetical populations would define the division that best fit the samples. The most probable division was into two groups; in the first group, the samples from individuals delivered in 2013 were most often segregated; moreover, in this year, a jump in inbreeding, expressed by the fixation index, was observed.

## 1. Introduction

The earliest evidence of falconry activity dates to around 5000 B.C. and comes from Tell Chuera, an archaeological site in northeastern Syria [1]. However, methods for breeding peregrine falcons in captivity were developed in the 1960s [2]. In Europe, there was a sharp decline in peregrine falcon populations in the 1950s and 1960s [3] caused largely by the widespread use of chlorinated hydrocarbon insecticides, mainly DDT (dichlorodiphenyltrichloroethane), in agriculture. The accumulation of these compounds led to the rubbing of falcon egg shells, resulting in eggs being crushed during hatching. This brought the species to the brink of extinction [4].

Reintroduction of the peregrine falcon was possible, thanks to intensive breeding efforts [2] Reintroduction is the release of representatives of the species into the environment, in areas that the species occupied in the past and where it became extinct. It assumes the establishment of a stable, self-sustaining population. Reintroduction programs have relatively low success rates [5]. However, using individuals from captivity, it has been possible to re-establish falcon populations in Sweden [6], France [7], Germany [8], the US [9] and the UK [10]. The reintroduction of specimens obtained from breeding is also used for other birds of prey, e.g., in efforts to restore the population of the common redshank (*Falco cherrug*) in Bulgaria [11].

In Poland, peregrine falcon reintroduction has been taking place since 1990 and focuses on restoring the tree-nesting ecotype population, with individuals from breeders located in Poland and Western European countries [2,12]. In 2010, the reintroduction program was taken over by the Falcon Wildlife Association, at which time the number of released birds increased significantly. Over the 2010–2015 period alone, 546 individuals were reintroduced, 201 more than over the 1990–2009 period when a total of 345 individuals were reintroduced. Between 2008 and 2019 (when our samples were collected), the following numbers of individuals were released into the environment: 2008: 23, 2009: 3, 2010: 56, 2011: 66, 2012: 75, 2013: 142, 2014: 129, 2015: 76, 2016: 38, 2017: 52, 2018: 78 and 2019: 55. Historically, the Polish population of peregrine falcons consisted mainly of representatives of the tree-nesting ecotype which occurred only in central and eastern Europe from northeastern Germany to central Russia [13,14]. The ecotype of falcons is defined from the nesting site; individuals of the tree-nesting ecotype nest in trees, while individuals of the urban ecotype nest on cliffs or mountain slopes and infrastructure elements resembling them (e.g., the tops of industrial chimneys). The influence of so-called “imprinting” is noted here, whereby birds, after leaving their parents’ nests, tend to nest in areas similar to those in which they were born. This mechanism perpetuates the division into ecotypes. It is also the reason that the restoration of the urban ecotype has not resulted in the natural restoration of the tree-nesting population [2]. The maintenance of two ecotypes inhabiting different environments can be very beneficial in terms of the conservation of the species. Both ecotypes belong to the same subspecies, *Falco peregrinus peregrinus* [12,15].

Nesje et al. [16] described a panel of genetic markers used for genetic variation analysis in peregrines and other falcons. These markers have been successfully used by other researchers, to describe among others, the genetic structure of the peregrine falcon [17], the Scandinavian peregrine population [18], wild and breeding populations of peregrine falcons and peregrines in the Czech Republic [3], and the Finnish peregrine population [19].

The aim of this study was to assess how the genetic structure of individuals used in reintroduction efforts has changed over the years 2008–2019.

## 2. Materials and Methods

### 2.1. Sampling and DNA Extraction

Peripheral blood was collected from wing veins via needle puncture, and then, stored in 96% ethanol (approval numbers 3181/2015 and 3445/2015). Blood samples were collected from 374 captive peregrine falcons. The samples were obtained from 47 breeders from 6 different countries—Poland (*n* =127), the Czech Republic (*n* = 117), Germany (*n* = 79), Slovakia (*n* = 38), Denmark (*n* = 10) and the Netherlands (*n* = 3)—prior to the birds being released into the wild as part of a polish restitution program. Samples were obtained during the years: 2008 (*n* = 11), 2010 (*n* = 46), 2011 (*n* = 53), 2012 (*n* = 73), 2013 (*n* = 65), 2014 (*n* = 43), 2015 (*n* = 35), 2016 (*n* = 6), 2017 (*n* = 16), 2018 (*n* = 18) and 2019 (*n* = 8). All individuals, divided by country, were used for the first part of the analysis; then, 170 individuals from the 6 breeders that provided the most falcons for reintroduction were analyzed. Of these breeders, 1 was from Germany (breeder 1), 1 was from Slovakia (breeder 3), 2 were from the Czech Republic (breeder 2 and breeder 5) and 2 were from Poland (breeder 4 and breeder 6).

DNA was extracted from blood using the NucleoSpin Tissue mini kit (Macherey-Nagel, Düren, Germany) in accordance with the manufacturer’s instructions.

### 2.2. Microsatellite Genotyping

Microsatellite genotyping was performed for all samples using 10 markers for which primers have been described in the literature for *Falco peregrinus* species [4,16,17,18] (GenBank sequence accession numbers AF118420-AF118434 [16]).

Based on the related literature, 10 markers were selected: NVHfp5 (labeled with VIC), NVHfp13 (labeled with 6-FAM), NVHfp46_1 (labeled with VIC), NVHfp54 (labeled with PET), NVHfp79_4 (labeled with 6-FAM), NVHfp82_2 (labeled with VIC), NVHfp86_2 (labeled with PET), NVHfp89 (labeled with 6-FAM), NVHfp92 (labeled with NED) and NVMfp107 (labeled with NED). These markers were characterized by the presence of high locus polymorphism, primer melting points and product length, which allowed the reactions to be carried out as one multiplex PCR (polymerase chain reaction) procedure. Reactions were performed as described by Puchała et al. [20]. Marker NVHfp79-4 was excluded from analysis due to null heterozygosity and low variability.

Visualization of the PCR product was performed using an ABI3500 DNA analyzer. For this purpose, the reaction product was mixed with formamide loading and size standard.

Allele sizes were assigned using GENEMAPPER 4.0 software, produced by Applied Biosystems Inc.

### 2.3. Statistical Analyses

Using GenAlEx v6.5 (Genetic Analysis in Excel) [21,22] software, genotypes were tested for departure from the Hardy–Weinberg equilibrium. All samples were pooled. Using GenAlEx and Excel (Microsoft) software, genotype comparisons, heterozygosity calculation and the estimation of allele numbers were performed. The probability of the occurrence of null alleles was estimated using Cervus 3.0.7 [23].

Relatedness and inbreeding analyses were performed using GenAlEx, and the fixation index for all analyzed samples and for groups which were analyzed in the second stage of the analysis were calculated. Data were visualized using Excel.

The genetic distance between the populations of the 6 largest breeders was estimated using Nei’s method [24] This method allows for the calculation of genetic distance based on allele frequencies and is implemented in GenAlEx. Phylogenetic trees were then constructed using the Neighbor-Joining method [25] in Mega X [26].

Simulation of the most probable division of individuals into groups was performed using STRUCTURE v2.3.4 [27,28,29,30] and STRUCTURE Harvester v0.6.94 [31]. First the Bayesian clustering method implemented in STRUCTURE software was used. This method uses allele frequencies calculated from multilocus genotypes and Monte Carlo Markov Chain (MCMC) sampling to assign individuals to given number of clusters (K). The assumption of the method is that there is the Hardy–Weinberg equilibrium in the given population [27]. Analyses were performed for K within the range of 1–19 in 3 repetitions for each value of K. The length of the burn-in period was set to 50,000, and the Number of MCMC Reps after burn-in was set to 500,000. Then, using STRUCTURE HARVESTER, the “Evanno” method was applied to the output from STRUCTURE. Evanno plots enable one to detect the number of K groups that best fits the data [32]. Specimens were separated by year for analysis in the structure program, then separate analyses were performed for specimens from a single breeder.

## 3. Results

### 3.1. All Samples

Multilocus genotypes were obtained in 10 loci. A total of 347 out of 374 individuals were genotyped in all loci, with 25 in nine loci and 2 in eight loci. Among the individuals genotyped at all loci, 85 had a unique genotype. No null alleles were found. The number of alleles observed at the loci ranged from four to nine. In one population, the private allele was present in the genotypes of 4 out of 38 individuals originating from Slovakia. The fixation index among all samples was 0.409. The genetic variability parameters across all the samples are presented in Table 1.

For groups divided by year of collection, Nei’s genetic distance varied from 0.009 (for the 2010–2011 pair) to 0.830 (for the 2008–2018 pair) with an average value of 0.280.

**Figure 1:**
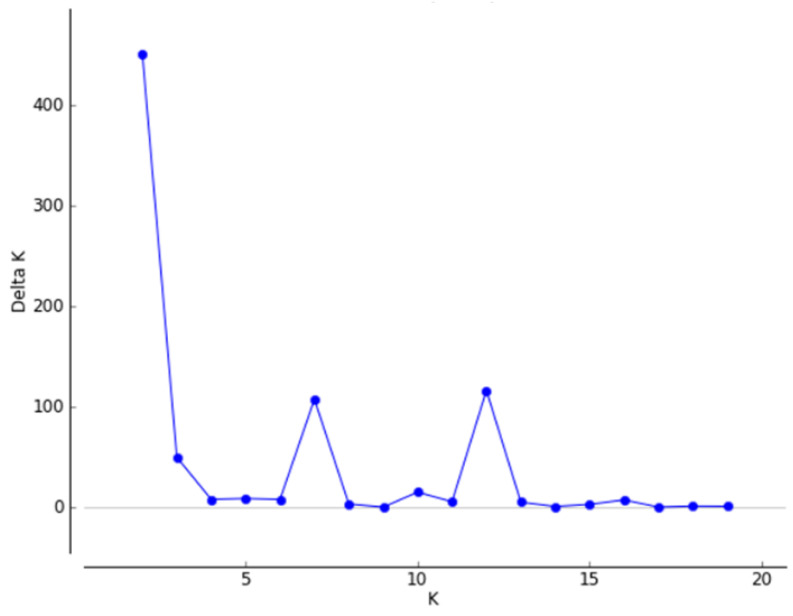
DeltaK plot showing the value of K (number of groups within population) that best fits the data.

**Figure 2:**
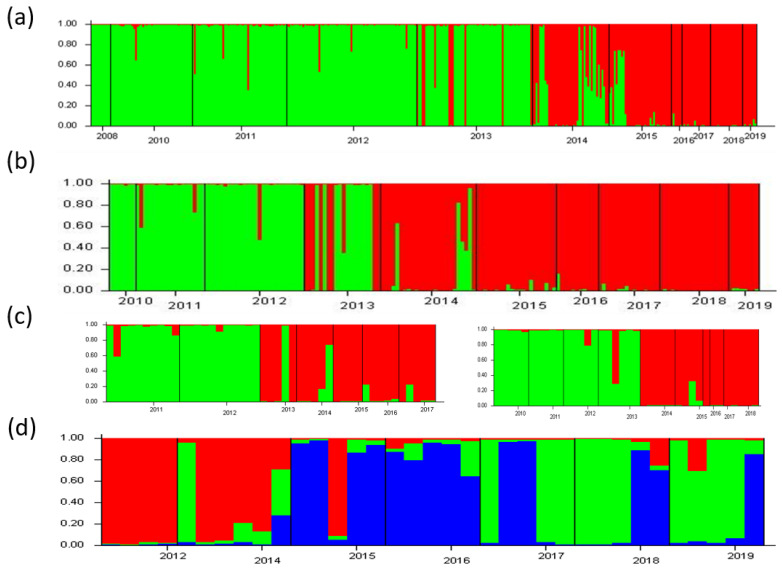
“STRUCTURE” plot that shows the genetic affiliation to groups computed by the program. Each column represents one individual and each color represents one genetic group. Individuals were divided by year of sampling. (**a**) All 374 individuals, K = 2; (**b**) 170 individuals from 6 main breeders, K = 2; (**c**) 45 individuals from breeder 1 and 38 individuals from breeder 3, K = 2; (**d**) 35 individuals from breeder 6, K = 3.

STRUCTURE analysis was performed for all the samples, divided by years. As presented in Figure 1, the most likely number of groups is K = 2. The “STRUCTURE” plot (Figure 2a) shows the change in the genetic structure of the birds between 2013 and 2014. Birds released after 2015 were mostly assigned to group 2 by the algorithm.

**Figure 3:**
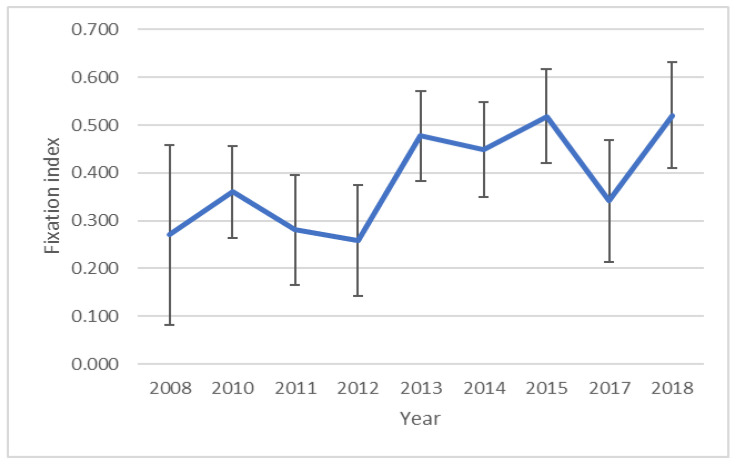
The plot shows the change in the average F (inbreeding) parameter over the years.

A fixation index (F) was used to determine the change in inbreeding over the years. The course of changes over the years is shown in Figure 3. A spike in mean inbreeding is evident between 2012 and 2013. The highest average inbreeding level was recorded for 2016; however, this is the year for which the fewest samples were collected.

### 3.2. Individuals from the Six Main Providers

Multilocus genotypes were obtained in nine loci (Marker NVHfp79-4 was excluded). A total of 163 out of 170 individuals were genotyped in all loci, 5 in no loci and 2 in seven loci, and 69 individuals had a unique genotype. No null alleles were found. The number of alleles observed at the locus ranged from four to nine. The fixation index among all samples was 0.409. The genetic variability factors across all samples are presented in Table 2.

**Figure 4:**
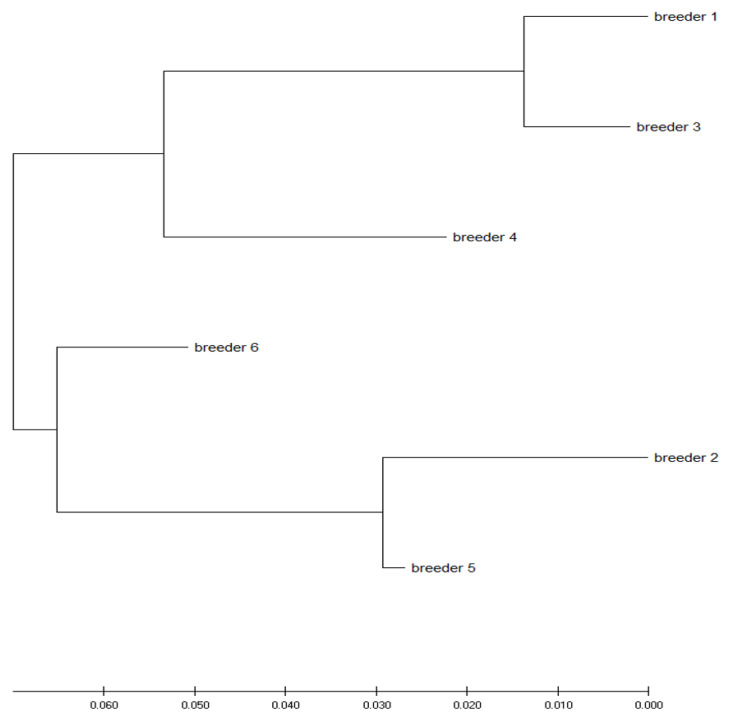
Nei’s distance relationship among breeding groups. The distances were inferred using the Neighbor-Joining method. The optimal tree with the sum of branch length = 0.19997414 is shown. The tree is drawn to scale, and its branch lengths use the same units as those of the evolutionary distances used to infer the phylogenetic tree.

On the phylogenetic tree, shown in Figure 4, two branches can be distinguished, on one of which are breeders 1, 3 and 4 (from Germany, Slovakia and Poland, respectively) and on the other, 2, 5 and 6 (from the Czech Republic, Czech Republic and Poland, respectively).

For the analysis of individuals from all six breeders, as for the analysis of all samples, the K = 2 groups best fit the data. In Figure 2b a change in genetic structure can be observed after the year 2013. The same trend is also visible in the analysis of all 374 individuals (Figure 2a). Figure 2c shows the changes in genetic structure of individuals from breeders 1 and 3. The plot in Figure 2d shows a 3-group split for individuals from breeder 6, as the analysis in STRUCTURE HARVESTER showed this to be the most likely split of individuals.

**Figure 5:**
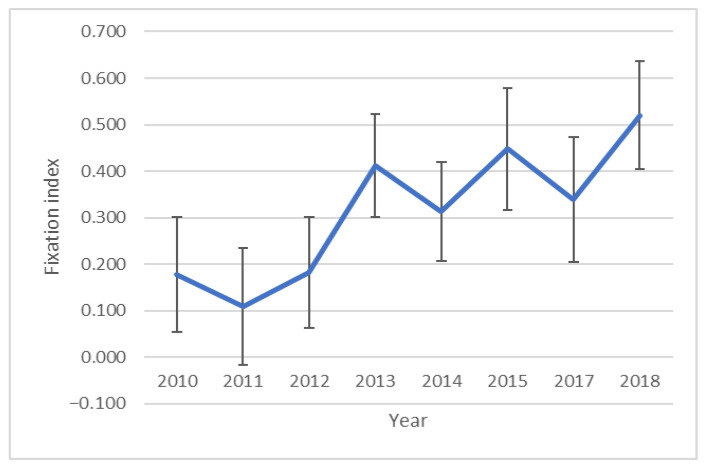
The change in mean F over the years for 6 main providers.

In both the analysis of all samples (Figure 3) and the analysis of the samples from the largest breeders (Figure 5), a large decrease in inbreeding in 2017 is noticeable.

## 4. Discussion

### 4.1. Variability in the Population

Compared to our previous study, which included both breeding and wild falcons [20], the number of observed alleles for two markers was lower (NVHfp107, NVHfp92_1) and for three was higher (NVHfp5, NVHfp54, NVHfp82_2). The effective number of alleles was lower for two markers (NVHfp13, NVHfp5) and higher for the other seven. The observed heterozygosity was lower for four markers (NVHfp13, NVHfp46_1, NVHfp86_2, NVHfp89) and higher for five.

Compared to the Finnish wild population, where 145 individuals sampled over 5 years were analyzed [19], for seven markers, the number of alleles was higher, for one it was lower, and one of the markers we used (NVHfp5) was not used in this study. The observed heterozygosity was lower for six markers (NVHfp13, NVHfp54, NVHfp82_2, NVHfp86_2, NVHfp89, NVHfp92-1) and higher for two markers (NVHfp107, NVHfp46_1).

Compared to the 32 (16 wild and 16 breeding) individuals from the Czech Republic [3], the number of alleles observed was lower for three markers (NVHfp107, NVHfp86-2 and NVHfp92-1) while it was higher for three others (NVHfp13, NVHfp5 and NVHfp54). Two markers were not used in the compared study (NVHfp46_1 and NVHfp82_2). In the captive population, the observed heterozygosity was lower for five markers (NVHfp107, NVHfp13, NVHfp5, NVHfp86_2,NVHfp92_1) and higher for two markers (NVHfp54, NVHfp89).

Based on the comparisons shown, it can be concluded that the captive falcon population shows signs of genetic diversity. The large standard deviation of the F-ratio seen in Figure 3 indicates that, most likely, the birds had a very diverse ancestry, which may have influenced the smaller annual increase in inbreeding.

Breeders from Poland are on both branches (Figure 4). Birds from these breedings are more similar to birds from foreign breedings. This is evidence that breeders, thanks to the open European market, are eager to obtain birds from abroad.

Despite the demonstrated genetic similarity between breeding pairs and their offspring for the selected breeders (Figure 4), it can therefore be concluded that falcon owners maintain good breeding practices, which is particularly important in the restitution process. The two different genetic groups, as is particularly evident in Figure 4, also contributed to the slower increase in inbreeding. The fact that peregrine falcon migration is counterintuitively not very large, probably due to the “imprinting” mechanism [2], means that wild populations of these birds do not show much genetic variability [16,17]. The system of breeding and of the exchange of birds used by falconers perhaps contributes more to the preservation of genetic variability than the natural exchange of generations combined with the dispersal of birds in heavily populated areas of Europe.

### 4.2. Variability among Years

In the case of the six breeders that have been supplying individuals most regularly over the years, a change in the genetic structure of the individuals supplied is also evident (Figure 2b). Additionally, in the case of birds from breeder 6, although the analyses indicated the most likely split in three groups, the change in structure is evident. Presented in Figure 5 is a notable change in the average inbreeding level between 2011 and 2013. The differences in the pattern of change from Figure 3 are probably due to the smaller number of samples and breeders.

The reason for the drop in inbreeding in 2017 (Figure 3 and Figure 5) may be the origin of the birds. The birds in question originated from four different breeding centers from four different countries: Germany (*n* = 5), Slovakia (*n* = 2), the Czech Republic (*n* = 4) and Poland (*n* = 5). Such different origins of the birds and the fact that these breeders, to our knowledge, do not cooperate with each other, may explain the decrease in inbreeding in 2017. In 2010, the year in which intensive restoration of the species began in Poland, the level of inbreeding, expressed by the fixation index, was relatively high (compared to, for example, the population analyzed by Mengoni et al. [33], in which the average fixation index was −0.031 (SE = 0.040)). The reason for this could be attributed to the foundations of breeding, which began in the mid-1960s when the species was already on the verge of extinction, so individuals used by falconers were allocated for intensive breeding [12]. The significant increase in inbreeding between 2013 and 2018 may be correlated with an increase in demand for falcons from breeding and being reintroduced. In 2010–2015, almost 550 birds were released under the Polish reintroduction program, much more than in the previous 20 years of its duration [12], such a large and constant demand for falcons forced an increase in the intensity of breeding; this is evident in the increase in inbreeding, and the shift over the years of the same is due to the time it took for falconers to develop their breeding regimes.

### 4.3. Possible Reasons for the Change in Genetic Structure

The change in the genetic structure of the samples studied is clearly visible in the structure charts (Figure 2) and occurs in late 2013 and early 2014. The demand for falcons is not high in Europe; buyers of the birds are enthusiasts, falconry hunters or bodies involved in the restoration of the species. The change in structure that we have observed has been evident since 2013/2014. Intensive reintroduction efforts in Poland began in 2010 [12], creating a relatively high and steady demand for falcons. This allowed the breeders involved in the project to expand their breeding operations and acquire new pairs. In addition, German breeders whose breeding operations had been extinguished after the successful reintroduction of the species were reactivated. This necessitated a greater exchange of birds between breeders and reaching out to new sources, such as the British Isles.

### 4.4. Conclusions

The idea of saving a wild species through breeding work is often criticized due to the apparently greater success of wild-to-wild translocations [5]. In the case presented in our study, it is thanks to the work of peregrine falcon breeders that a basic reintroduction population has been established, which is characterized (compared to published data to date), by good genetic parameters. The future will show to what extent the described structure will be modified in the free-living population.

The data we collected suggest an impact of increased demand for birds, associated with reintroduction, on the genetic structure of birds kept in the breeding facilities from which the birds originated. This is a factor that should be taken into account in reintroduction plans, especially when the planned activities are intensive and extend over time.

## Figures and Tables

**Table 1:** Analysis of genetic variability for all samples.

Locus	Allele Ranges	N	Na	Ne	Ho	He	F
NVHfp107	203–208	373	4	2.082	0.466	0.520	0.102
NVHfp13	93–103	373	9	4.654	0.488	0.785	0.379
NVHfp46_1	117–122	373	6	4.129	0.539	0.758	0.289
NVHfp5	102–108	374	5	1.462	0.070	0.316	0.780
NVHfp54	104–208	369	9	2.487	0.428	0.598	0.284
NVHfp82_2	134–140	373	5	1.648	0.137	0.393	0.652
NVHfp86_2	140–145	368	5	3.453	0.351	0.710	0.507
NVHfp89	116–132	367	9	4.831	0.569	0.793	0.282
NVHfp92_1	110–126	365	7	3.011	0.288	0.668	0.569

N—number of scored individuals, Na—observed number of alleles, Ne—effective number of alleles, Ho—observed heterozygosity, He—expected heterozygosity, F—fixation index.

**Table 2:** Analysis of genetic variability for 6 main providers samples.

Locus	Allele Ranges	N	Na	Ne	Ho	He	F
NVHfp107	203–208	170	4	1.951	0.465	0.488	0.047
NVHfp13	96–103	169	8	4.161	0.349	0.760	0.540
NVHfp46_1	117–122	170	6	3.886	0.494	0.743	0.335
NVHfp5	102–108	170	4	1.709	0.082	0.415	0.801
NVHfp54	104–115	170	6	2.425	0.441	0.588	0.249
NVHfp82_2	134–140	169	5	2.509	0.195	0.602	0.675
NVHfp86_2	140–145	168	5	3.716	0.315	0.731	0.568
NVHfp89	116–132	167	9	5.598	0.575	0.821	0.300
NVHfp92_1	110–124	168	6	3.4051	0.310	0.706	0.562

N—number of scored individuals, Na—observed number of alleles, Ne—effective number of alleles, Ho—observed heterozygosity, He—expected heterozygosity, F—fixation index. For groups divided by year of collection, Nei’s genetic distance varied from 0.025 (for the breeder 1–breeder 3 pair) to 0.160 (for the breeder 1–breeder 2 pair) with an average value of 0.087.

## Data Availability

The data presented in this study are available on request from the corresponding author.
